# NGF-induced TrkA/CD44 association is involved in tumor aggressiveness and resistance to lestaurtinib

**DOI:** 10.18632/oncotarget.3227

**Published:** 2015-03-25

**Authors:** Léo Aubert, Matthieu Guilbert, Cyril Corbet, Elisabeth Génot, Eric Adriaenssens, Thierry Chassat, François Bertucci, Thomas Daubon, Nicolas Magné, Xuefen Le Bourhis, Robert-Alain Toillon

**Affiliations:** ^1^ INSERM U908, 59655 Villeneuve d'Ascq, France; ^2^ University Lille 1, 59655 Villeneuve d'Ascq, France; ^3^ SIRIC OncoLille, 59000 Lille, France; ^4^ INSERM U1045, 33607 Pessac, France; ^5^ CNRS UMR 8161, 59000 Lille, France; ^6^ PLETHA, Institut Pasteur de Lille, 59000 Lille, France; ^7^ INSERM UMR 891, Institut Paoli-Calmettes, 13009 Marseille, France; ^8^ Radiobiologie Cellulaire et Moléculaire, EMR3738 - Equipe 4, Faculté de Médecine Lyon-Sud, 69000 Lyon, France; ^9^ Département de Radiothérapie, Institut de Cancérologie Lucien Neuwirth, 42270 Saint Priest en Jarez, France

**Keywords:** CD44, NGF, TrkA, lestaurtinib, resistance

## Abstract

There is accumulating evidence that TrkA and its ligand Nerve Growth Factor (NGF) are involved in cancer development. Staurosporine derivatives such as K252a and lestaurtinib have been developed to block TrkA kinase signaling, but no clinical trial has fully demonstrated their therapeutic efficacy. Therapeutic failures are likely due to the existence of intrinsic signaling pathways in cancer cells that impede or bypass the effects of TrkA tyrosine kinase inhibitors. To verify this hypothesis, we combined different approaches including mass spectrometry proteomics, co-immunoprecipitation and proximity ligation assays. We found that NGF treatment induced CD44 binding to TrkA at the plasma membrane and subsequent activation of the p115RhoGEF/RhoA/ROCK1 pathway to stimulate breast cancer cell invasion. The NGF-induced CD44 signaling was independent of TrkA kinase activity. Moreover, both TrkA tyrosine kinase inhibition with lestaurtinib and CD44 silencing with siRNA inhibited cell growth *in vitro* as well as tumor development in mouse xenograft model; combined treatment significantly enhanced the antineoplastic effects of either treatment alone. Altogether, our results demonstrate that NGF-induced tyrosine kinase independent TrkA signaling through CD44 was sufficient to maintain tumor aggressiveness. Our findings provide an alternative mechanism of cancer resistance to lestaurtinib and indicate that dual inhibition of CD44 and TrkA tyrosine kinase activity may represent a novel therapeutic strategy.

## INTRODUCTION

Nerve growth factor (NGF), the first growth factor discovered by Stanley Cohen and Rita Levi Montalcini [1], functions in both normal tissues and cancers of different origins [2]. In particular, NGF is produced and released by breast cancer and tumor-associated stromal cells. Its expression is known to drive angiogenesis [3] and to be responsible for bone metastasis pain [4]. TrkA, the membrane receptor tyrosine kinase (RTK) of NGF, plays an important role in NGF-mediated biological effects. TrkA expression is associated with perineural invasion of cancer cells [5]. Mounting evidence suggests that NGF not only affects tumor-associated cells but also dictates cancer cell behaviors [6]. Interestingly, in breast cancer cells, TrkA activation is correlated with the high pro-invasive and metastatic potential [7–9] that is associated with poor clinical outcomes [10, 11]. Tyrosine kinase inhibitors derived from staurosporine (K252a, lestaurtinib) are reported to exert antitumor effects in both *in vitro* and preclinical *in vivo* models [12]. Nevertheless, TrkA kinase inhibitors failed to demonstrate therapeutic efficacy in clinical trials [13]. The lack of objective responses to TrkA inhibitors in clinical trials has been linked to insufficient bioavailability of the drugs [14] and more recently, to an intrinsic resistance mechanism in cancer cells involving NF-kappa B signaling [15]. Interestingly, tyrosine kinase inhibitor effects may be also due to interactions with other membrane receptors as exemplified by the interaction of numerous RTKs with CD44 [16]. CD44 is a cell surface glycoprotein encoded by a single 20-exons gene that generates a standard form (CD44s) and more than twenty variant isoforms (CD44v) [17]. CD44 is also known as a marker of cancer stem cells [18] and its expression may cause therapeutic failure in many cancers [19].

In this report, we demonstrate for the first time that NGF induces TrkA/CD44 interaction independent of TrkA phosphorylation in cancer cells. The subsequent CD44 downstream signaling is implicated in cancer cell invasion and growth. Altogether, our findings provide an alternative mechanism of cancer resistance to lestaurtinib and indicate that dual inhibition of CD44 and TrkA tyrosine kinase activity may represent a novel therapeutic strategy.

## RESULTS

### CD44 association with TrkA at the plasma membrane is enhanced by NGF stimulation

**Figure 1:**
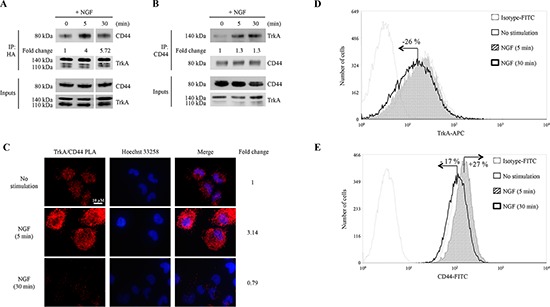
NGF induces binding of CD44 to Trk AHA-TrkA MDA-MB-231 cells were treated with NGF (5 and 30 min). **(A)** CD44 and TrkA complexes were detected by IP with anti-HA antibody followed by immunoblotting with anti-CD44 antibody. CD44 binding to HA-TrkA was normalized and indicated as Fold Change. CD44 binding to HA-TrkA at 0 min was considered as 1. **(B)** Reverse IP with anti-CD44 antibody followed by immunoblotting with anti-HA antibody. HA-TrkA binding to CD44 was normalized and indicated as Fold Change. HA-TrkA binding to CD44 at 0 min was considered as 1. **(C)** TrkA/CD44 association was visualized by a PLA; interactions between TrkA and CD44 are shown as red spots. Density of red spots was quantified by Image J software. TrkA/CD44 PLA density without stimulation was considered as 1. **(D and E)** Flow cytometry analyses of membrane levels of TrkA (D) and CD44 (E) Fold Changes were determined using the median value of each histogram.

MDA-MB-231 breast cancer cells overexpressing HA-TrkA were used to examine the signaling pathways involved in NGF response as described previously [8]. Cells were treated with NGF for 30 min, cell lysates were then subjected to HA immunoprecipitation (IP) or streptavidin pull-down. Eluates were resolved by SDS-PAGE and visualized with colloidal Coomassie Blue staining (Supplementary Figure S1A) and classified with gene ontology (Supplementary Figure S1B). Several bands of increased intensity were detected in NGF-treated cells using IP corresponding to proteins that may be specifically bound to TrkA. These bands were excised for identification using mass spectrometry. Among the proteins identified, we found CD44 and several of its known signaling partners including Ezrin, Moesin, p115RhoGEF (Rho guanine nucleotide exchange factor 1), FAK1 (Focal adhesion kinase 1), ARP2C (Actin-related protein 2/3 complex subunit 2), Alpha-actinin-1, LIMA1 (LIM domain and actin-binding protein 1) (Table 1). We then performed biotin labeling and streptavidin pull-down to analyze NGF-induced potential modifications of membrane associated proteins. Several bands of increased intensity upon NGF stimulation were observed (Supplementary Figure S1) and processed for mass spectrometry analysis. The following proteins were identified: RhoA (Transforming protein RhoA), RhoC (Rho-related GTP-binding protein C), Guanine nucleotide-binding protein subunit alpha-13, Arp2 (Actin-related protein 2), R-Ras2 (Ras-related protein 2), Basigin (CD147) and Actin 2 (Actin cytoplasmic 2) (Table 1). There is increasing evidence that CD44 is involved in cancer development [19], but it has not been reported to interact with TrkA or participate in its downstream signaling. To validate the interaction between TrkA and CD44, we performed IP and reverse IP, using HA and CD44 antibodies, respectively (Figure 1A and 1B). TrkA and CD44 were co-immunoprecipitated (co-IP) in NGF-untreated control cells, and the co-IP was further increased upon NGF treatment. We then performed a proximity ligation assay (PLA) to determine any direct interaction between CD44 and TrkA at the plasma membrane (Figure 1C). In the absence of NGF, a clear PLA signal (red dots) was observed indicating that even without stimulation TrkA binds CD44. When cells were treated with NGF for 5 min, a 3-fold increase in the TrkA/CD44 complexes was observed at the plasma membrane. This increase was transient as the PLA signal decreased to basal level after 30 min of NGF treatment. The observed kinetics of CD44/TrkA association might be due to TrkA shuttling as PLA experiments were performed without membrane permeabilization. Indeed, flow cytometry analysis showed that membrane TrkA was not significantly altered after 5 min of NGF treatment, but was reduced after 30 min (about 25%), indicating that TrkA may be internalized (Figure 1D). Membrane CD44 was also increased (27%) after 5 min of NGF treatment but decreased under basal levels after 30 min (−17%) (Figure 1E). TrkA/CD44 association was also tested in cell lines from breast, prostate, colon, head and neck cancers (Supplementary Figure S2) although total expression levels of CD44 and TrkA varied between the cell lines (Supplementary Figure S2A). In PC3 prostate cancer cells, which express relatively high levels of both CD44 and TrkA, NGF stimulation resulted in the association of TrkA with CD44 at the plasma membrane, as demonstrated using the PLA signal of TrkA/CD44 (Supplementary Figure S2B). Altogether, these results indicate that NGF induces association between TrkA and CD44 at the plasma membrane in cancer cells of various origins.

**Table 1:** Identification of putative CD44 signaling partners under NGF stimulation Mass spectrometry identification of putative interacting partners of TrkA (HA-TrkA IP) and plasma membrane-associated proteins (streptavidin pull-down) under NGF stimulation was performed as described in materials and methods.

	Protein name	Uniprot ID	Mass (kDa)	Number of peptides	Mascot score	Refs
**TrkA-interacting proteins (HA-TrkA immunoprecipitation)**	CD44 antigen	P16070	81	3	125	[19]
	Ezrin	P15311	80	22	318	[38]
	Moesin	P26038	75	9	165	[39]
	Rho guanine nucleotide exchange factor 1	Q92888	115	11	104	[20, 40]
	Focal adhesion kinase 1	Q05397	125	2	40	[41]
	Actin-related protein 2/3 complex subunit 2	O15144	34	8	122	[42, 43]
	Guanine nucleotide-binding protein subunit beta-2-like 1	P63244	35	20	578	[44]
	Alpha-actinin-1	P12814	103	20	245	[45]
	LIM domain and actin-binding protein 1	Q9UHB6	85	3	64	[45]
**Plasma membrane-associated proteins (streptavidin pull-down)**	Transforming protein RhoA	P61586	21	6	204	[46, 47]
	Rho-related GTP-binding protein RhoC	P08134	22	6	219	[46, 47]
	Guanine nucleotide-binding protein subunit alpha-13	Q14344	44	10	133	[48]
	Actin-related protein 2	P61160	44	16	326	[43]
	Basigin	P35613	42	8	182	[49]
	Ras-related protein R-Ras2	P62070	23	3	62	[50]
	Actin, cytoplasmic 2	P63261	42	21	569	[45]

### CD44 diversifies NGF signaling through the RhoA/RhoC/ROCK1 pathway to stimulate cell invasion

**Figure 2:**
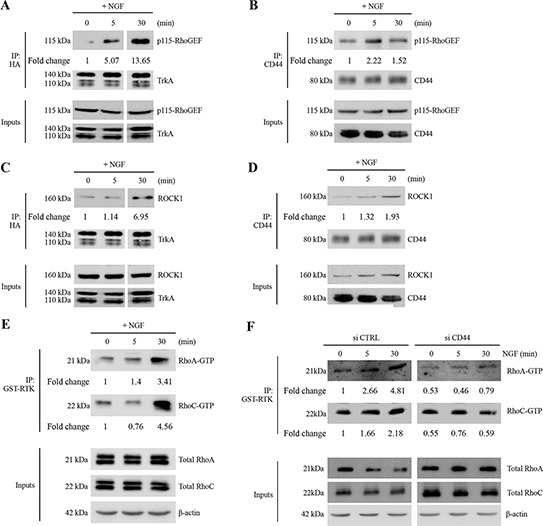
NGF induces association of TrkA with CD44/p115RhoGEF/RhoA/RhoC/ROCK1 and RhoGTPase activation **(A–D)** NGF induces TrkA association with CD44/p115RhoGEF/RhoGTPases/ROCK1. HA-TrkA MDA-MB-231 cells were treated with NGF (5 and 30 min). IPs were done with anti-HA or anti-CD44 antibodies, and immunoblotting was used to detect the presence of p115RhoGEF (A and B) and ROCK1 (C and D) in the eluate. **(E)** NGF increases RhoGTPase activity. HA-TrkA MDA-MB-231 cells were stimulated with NGF (5 or 30 min) and GTP-bound RhoA and RhoC were determined in cell lysates by an affinity pull-down assay with the GST-Rho binding domain followed by immunoblotting for RhoA and RhoC. Whole cell lysate samples were immunoblotted for total RhoA/C as a control. Figures are representative of three independent pull-down assays. **(F)** CD44 invalidation inhibits RhoA and RhoC activities. HA-TrkA MDA-MB-231 cells were transfected with siCTRL (scramble siRNA) or siCD44 stimulated with NGF (5 or 30 min) and GTP-bound RhoA and RhoC were determined in cell lysates by an affinity pull-down assay with the GST-Rho binding domain followed by immunoblotting for RhoA and RhoC. Whole cell lysate samples were immunoblotted for total RhoA/C as a control.

**Figure 3:**
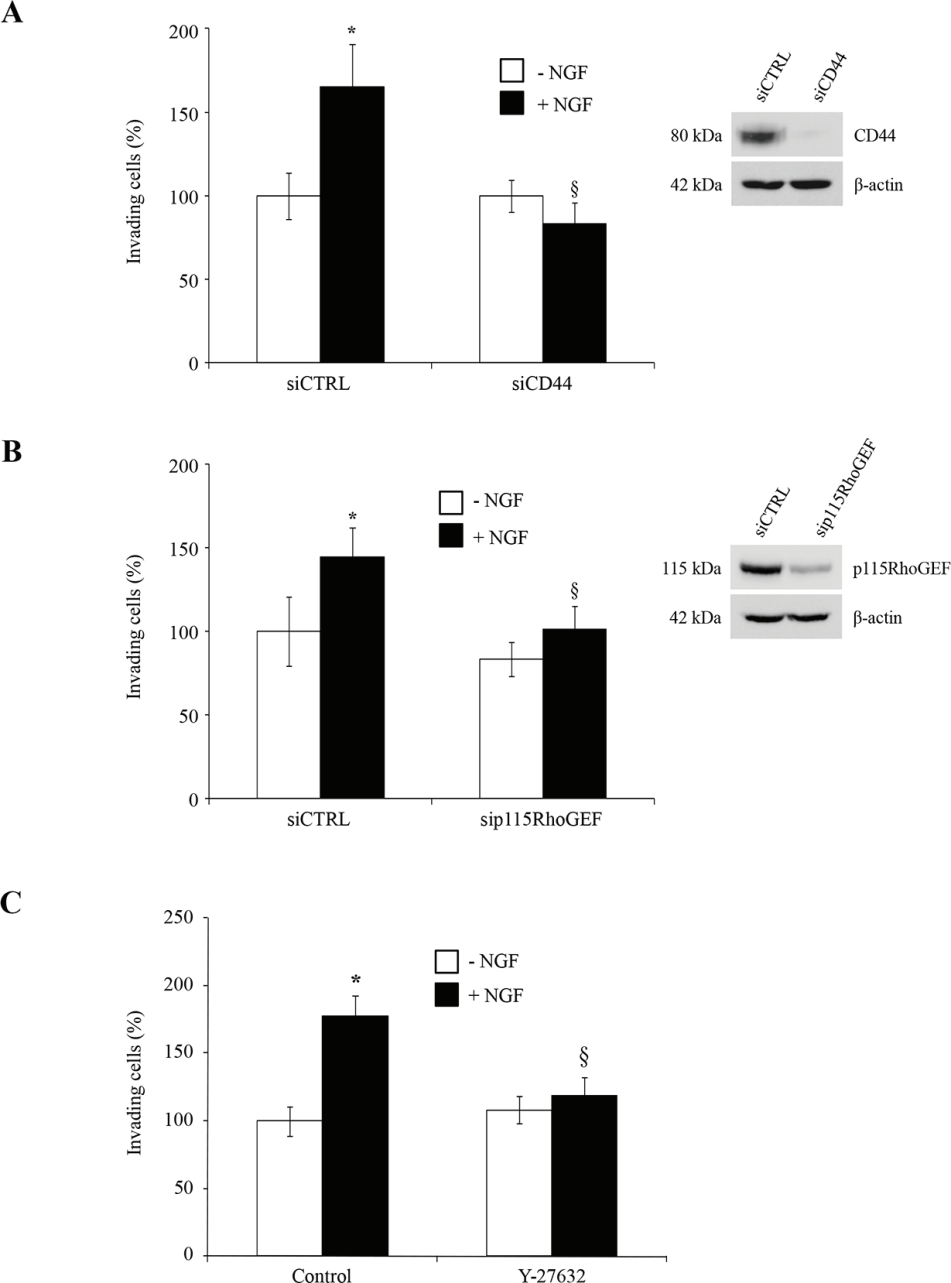
CD44, p115RhoGEF and ROCK1 are involved in NGF-stimulated cell invasion HA-TrkA MDA-MB-231 cells were transfected with si CTRL (scramble siRNA), siCD44 (A) or sip115RhoGEF (B). ROCK1 involvement in cell invasion was assessed using the Y-27632 compound (C). HA-TrkA MDA-MB-231 cells were treated with NGF and invading cells were evaluated using Transwells. Data are mean ± S.D. of three experiments done in triplicate and are presented as a percentage of controls. Statistical analysis was performed with one-way ANOVA followed by Bonferroni's post-test. Error bars represent S.D. *p < 0.001 for NGF stimulation *versus* no stimulation; § *p* < 0.001 for experimental *versus* control under NGF stimulation. siRNA efficiency was assessed by immunoblot with specific antibodies against CD44 or p115RhoGEF. β-actin was used as a loading control.

CD44 is known to stimulate breast tumor cell invasion through the RhoA pathway [20]. Interestingly, we found that p115RhoGEF co-immunoprecipitated with TrkA in NGF-treated cells (Table 1). To confirm the downstream association of p115RhoGEF to the TrkA/CD44 complex, we used co-IP to investigate whether they directly interact. Indeed, antibodies against the HA tag or CD44 were able to pull down p115RhoGEF (Figure 2A and 2B). Moreover, upon NGF treatment, ROCK1 (the main target of RhoA and RhoC) was also found to co-IP with TrkA and CD44 (Figure 2C and 2D). The involvement of p115RhoGEF and ROCK1 in NGF-induced signaling was further supported by the activation of RhoA and RhoC, which are p115RhoGEF downstream targets (Figure 2E). In order to ascertain that CD44 is responsible of RhoGTPase activation, we measured RhoA and RhoC activities after CD44 invalidation by siRNA. As shown in Figure 2F, CD44 siRNA reduced RhoGTPAse activities for both RhoA and RhoC. We also analyzed the invasive capacity of breast cancer cells *in vitro* using a transwell system (Figure 3A–3C). We found that inhibition of CD44 and p115RhoGEF either through siRNA or the ROCK1 specific inhibitor Y-27632 totally abolished NGF-induced invasion Interestingly, blocking CD44 affected neither NGF-induced TrkA phosphorylation nor the canonical TrkA pathways that signal through Akt and Src (Supplementary Figure S3). Together, these results indicate that the TrkA/CD44 complex induced by NGF is able to activate the RhoGTPAse signaling pathway to enhance cell invasion, independently of TrkA canonical pathways.

### TrkA/CD44 association is independent of NGF-induced TrkA phosphorylation

**Figure 4:**
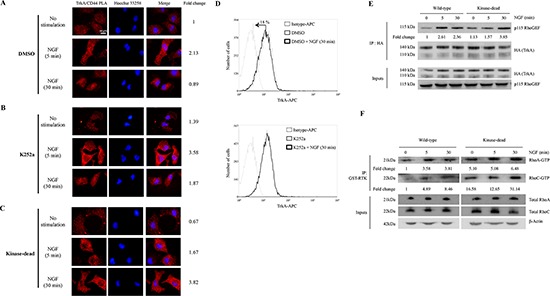
TrkA binds to CD44 independently of its kinase domain activity **(A–C)** HA-TrkA MDA-MB-231 cells were incubated with DMSO (A) or with K252a (B) HA-TrkA kinase-dead MDA-MB-231 cells (C) were treated with NGF (5 or 30 min). The interactions between TrkA and CD44 were visualized by the detection of red spots by PLA. Density of red spots was quantified by Image J software. TrkA/CD44 PLA density without stimulation in DMSO was considered as 1. **(D)** K252a impedes TrkA internalization as monitored by flow cytometry. Results are representative of three independent experiments. **(E)** HA-TrkA (wild-type) and HA-TrkA Kinase-dead expressing cells were treated with NGF (5 and 30 min). IPs were done with anti-HA and immunoblotting was used to detect the presence of p115RhoGEF. P115RhoGEF binding to HA-TrkA was normalized and indicated as Fold Change. P115RhoGEF binding to HA-TrkA at 0 min was considered as 1. **(F)** GTP-bound RhoA and RhoC were determined in cell lysates by an affinity pull-down assay with the GST-Rho binding domain followed by immunoblotting for RhoA and RhoC. Whole cell lysate samples were immunoblotted for total RhoA/C as a control.

Our findings that blocking CD44 abolished NGF-induced cell invasion but did not affect NGF-induced TrkA tyrosine kinase phosphorylation, prompted us to evaluate whether the activation state of TrkA could affect the TrkA/CD44 association at the plasma membrane. We performed a PLA on cells treated with TrkA tyrosine kinase inhibitor K252a or on cells expressing TrkA kinase-dead mutant to inhibit TrkA phosphorylation (Supplementary Figure S4). A 2-fold increase of PLA signal was observed at the plasma membrane after 5 min of treatment with NGF, the PLA signal intensity returned to basal levels after 30 min of treatment (Figure 4A). Similar results were observed in both K252a-treated cells (Figure 4B) and cells expressing TrkA kinase-dead mutant (Figure 4C) after 5 min of treatment with NGF. These results indicated that the interaction between TrkA and CD44 is independent of TrkA tyrosine kinase activity. However, after 30 min of NGF treatment, TrkA/CD44 interaction did not return to basal levels in K252a-treated (Figure 4B) and kinase-dead mutant (Figure 4C) cells compared to control (Figure 4A). This could be due to suboptimal TrkA internalization as it has been reported that phosphorylation of TrkA offers docking sites for cellular endocytosis machinery [21]. To verify this possibility, we performed flow cytometry analysis to evaluate the membrane levels of TrkA (Figure 4D). Indeed, after 30 min of NGF treatment, no diminution of TrkA levels was observed in cells treated with K252a compared with DMSO-treated control cells, indicating that inhibition of TrkA phosphorylation slowed TrkA internalization. We then measured p115RhoGEF binding to TrkA and RhoGTPase activation in TrkA kinase dead mutant cells. As shown in Figure 4E and 4F, p115RhoGEF binding to TrkA was still enhanced under NGF treatment (3.8 fold increase after 30 min of NGF treatment) (Figure 4E). Moreover, in TrkA kinase-dead cells, RhoA and RhoC were activated in absence of NGF treatment and NGF further increased these activities. Together, these results indicate that TrkA/CD44 association and the subsequent RhoGTPase activation are not abolished by the inhibition of TrkA phosphorylation. In contrast, the inhibition of TrkA phosphorylation seems to maintain TrkA/CD44 complex.

### Combinational targeting of TrkA and CD44 exhibits enhanced anti-tumor activity

**Figure 5:**
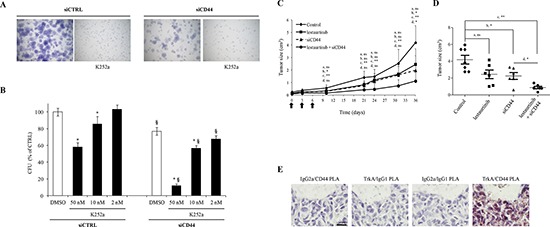
Effects of combined targeting of TrkA and CD44 on tumor cell growth **(A and B)** Effects of siCD44 and TrkA kinase inhibition by K252a on colony formation. Cells were plated at single cell density and cultured for one week. (A) Representative pictures of forming colonies. (B) Quantification of colony forming units (CFU). **(C and E)** Impact of TrkA or/and CD44 inhibitions on tumor growth *in vivo*. Xenograft experiments were conducted using HA-TrkA MDA-MB-231 cells. The tumors were allowed to develop for 14 days and the mice were then submitted to 3 injections (every 3 days; black arrows) of either *in vivo* Jet PEI + scramble siRNA (7.5 μg/mouse, Control), lestaurtinib (10 mg/kg), Jet PEI + siCD44 (7.5 μg/mouse) or lestaurtinib and Jet PEI + siCD44 (7.5 μg/mouse). (C) Tumor volumes measured at different intervals. A Mann-Whitney test was performed between the control group and lestaurtinib (a), the control group and siCD44 (b), the control group and lestaurtinib + siCD44 (c), and between the siCD44 group and lestaurtinib + siCD44 (d). *P < 0.05; **P < 0.01; ns: not significant. **(D)** Dot plot representation of tumor volume at the end of the experiment. **(E)** Brightfield PLA assays of TrkA/CD44 interaction on MDA-MB-231 tumor xenografts. PLA assays were performed on tumor xenografts from control group. Pictures are representative of three independent PLA assays.

As the formation of TrkA/CD44 complex was independent of TrkA phosphorylation, and TrkA tyrosine inhibitor K252a allowed for sustained TrkA and CD44 association at the plasma membrane, we wanted to know if combinational targeting of TrkA and CD44 could more efficiently inhibit tumor cell growth. As shown in Figure 5A and 5B, K252a treatment of MDA-MB-231 cells reduced both the size and the number of colonies. Blocking CD44 by siRNA only slightly decreased colony formation. Interestingly, combined treatment of cells with K252a and siCD44 dramatically reduced clonogenic cell growth. We next evaluated the efficiency of these treatments *in vivo* by performing tumor xenograft experiments in the SCID mouse model. As shown in Figure 5C and 5D, lestaurtinib (clinical derivative of K252a) or siCD44 reduced tumor growth when compared to control (scramble siRNA). Combined treatment of lestaurtinib and siCD44 resulted in a dramatic reduction of tumor burden when compared to lestaurtinib or siCD44 treatment alone. We also confirmed the direct interaction between TrkA and CD44 in tumor xenografts using a PLA (Figure 5E).

## DISCUSSION

**Figure 6:**
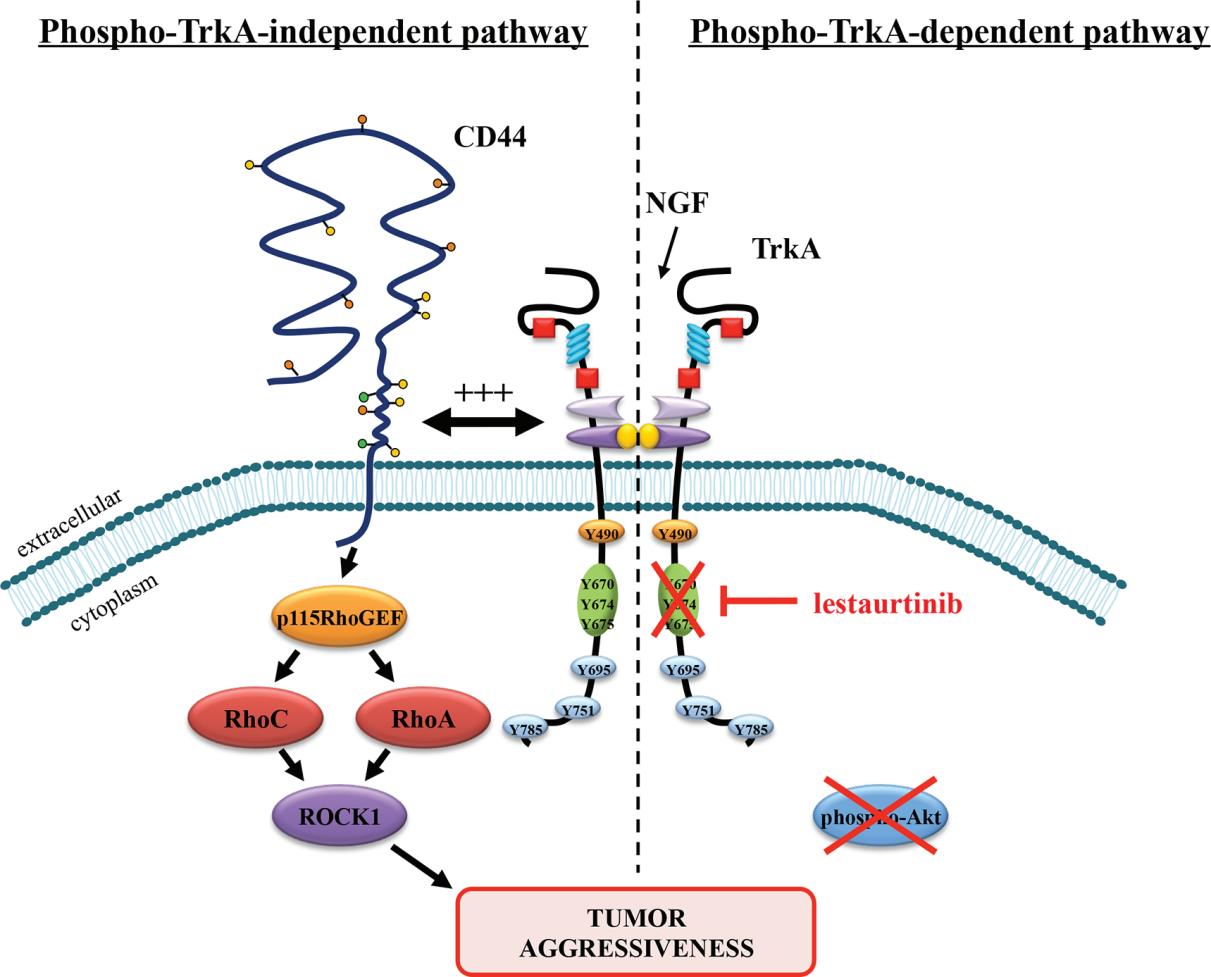
Proposed model of lestaurtinib resistance through a phospho-TrkA-independent pathway involving downstream CD44 signaling Lestaurtinib inhibits the kinase activity of TrkA and phospho-TrkA-dependent downstream signaling including Akt. However, a phospho-TrkA-independent pathway that uses CD44 signaling may serve as an alternative pathway to strengthen tumor aggressiveness and to escape lestaurtinib inhibition.

In this study, we report for the first time that TrkA is associated with CD44 in cancer cells. CD44 is the principal receptor for the large glycosaminoglycan hyaluronan. CD44 lacks kinase activity but influences cell behaviors such as uncontrolled growth, apoptosis evasion, angiogenesis, cell motility and invasion through various mechanisms [22]. CD44 directly interacts with key intracellular regulators of the actin cytoskeleton such as ankyrin, and members of the ezrin, radixin, and moesin (ERM) protein families. CD44 can also function as a co-receptor to modulate signaling from a diverse set of membrane receptors including integrins, multidrug resistance complex components (MDR 1, CD147) and growth factor receptors such as EGFR, PDGFR, FGFR, and c-Met [19]. CD44 cooperates with RTKs through different mechanisms. For instance, CD44 transactivates the ErbB2/ErbB3 heterodimer by Src-mediated phosphorylation of ErbB2. CD44 can also initiate growth factor receptor signaling by interacting with various ligands. For instance, binding of proHBEGF to CD44v3 induces cleavage of the proform and release of HBEGF that in turn activates ErbB4. CD44v also binds to HGF/SF and FGF and presents them to their specific RTK [23]. A splice variant of CD44 expressed in the apical ectoderm ridge presents FGFs to limb mesenchyme and is required for limb outgrowth [24]. In our cell models, because CD44 is co-immunoprecipitated with TrkA and we observed a positive signal from the PLA, a direct association between TrkA and CD44 is suggested. Alternatively, NGF is known to bind to heparan sulfate with low affinity [25], it is possible that NGF serves as a bridge between TrkA and CD44. Moreover, the formation of TrkA/CD44 complex upon NGF stimulation leads to the activation of the canonical CD44 pathway involving p115RhoGEF, RhoA and ROCK1 (Figure 6). TrkA and CD44 co-expression has been already reported in neuroblastoma cells [26] but the authors did not examine their interaction nor the resulting intracellular signaling. TrkA signaling has been widely described in nerve systems and similar signaling pathways have also been found in cancer cells. Indeed, NGF binding to TrkA induces phosphorylation of TrkA, phospho-TrkA then recruits various intracellular adaptors (e.g. Shc, Grb2, or PI3K) to activate MAPK or PI3K, resulting in cell growth or differentiation depending on the cell type. TrkA signaling is also modulated by several co-receptors such as p75^NTR^, GPCR (G protein-coupled receptor), and Ret-5. P75^NTR^ is a low affinity receptor and is the first described co-receptor of TrkA. After its binding to p75^NTR^, NGF has an increased affinity for TrkA [27]. In addition, p75^NTR^ also delays TrkA ubiquitination and sustains its phosphorylation [28]. However, we have previously shown that p75^NTR^ is not involved in NGF-stimulated invasion of breast cancer cells [8]. Transactivation of TrkA receptors in PC12 cells and TrkB in hippocampal neurons has been observed after treatment with adenosine or PACAP neuromodulators, both of which act through GPCRs [29]. Trk receptor transactivation by adenosine or PACAP requires a longer time course. Furthermore, the increase in Trk activity can be inhibited by the use of K252a, PP1 and Src family-specific inhibitors. Trk receptors also have the capacity to activate the Ret-51 receptor tyrosine kinase in postnatal superior cervical ganglion (SCG) neurons. Ret-51 activation does not depend on the PI3K or MAPK pathways and occurs with a very slow kinetics, similar to GPCR activation of Trk receptors [30].

Receptor tyrosine kinases are widely activated in cancers, and have been the focus of targeted therapies for several decades. TrkA is a prototypical RTK and phospho-TrkA has been correlated with the tumor aggressiveness in breast cancer [7]. We showed that in breast cancer cell lines, the biological effects of TrkA are mainly supported by its phosphorylation [8, 9]. Moreover, it has been found that NTRK1 fusion or rearrangement that constitutively activates TrkA by phosphorylation is oncogenic in lung tissue [31]. Altogether, these data indicate the importance of phospho-TrkA and its canonical signaling pathways in tumor development and suggest that tyrosine kinase inhibitors would be good candidates for cancer therapy. Nevertheless, despite the powerful anti-tumor effect *in vitro* and *in vivo* [32–34], therapeutic benefits of such compounds have not been documented. In this report, we show that the NGF/TrkA axis engages CD44 as a co-receptor to strengthen cancer cell invasion and growth. This interaction is independent of the kinase activity of TrkA. Our findings provide a rational for the development of new therapeutics by simultaneously blocking both the kinase dependent and independent effects of TrkA.

## METHODS

### Cell culture

The MDA-MB-231 breast cancer cell line was obtained from the American Type Culture Collection (ATCC). MDA-MB-231 overexpressing HA-TrkA or kinase-dead TrkA were established and maintained in culture as previously described [8]. Cells were amplified and passaged no more than 25 times. Before treatment, cells were rinsed twice with PBS, left for 24 h in culture medium supplemented with 0.1% fetal bovine serum (FBS), then treated with recombinant human β-NGF (denoted as NGF and used at a 16 nM concentration in all experiments) (Scil Proteins, Germany). For some experiments, cells were pre-incubated for 1 h with the pharmacological inhibitors K252a (10 nM) or Y-27632 (10 μM) (Calbiochem, UK) before NGF treatment.

### Cell extracts

Subconfluent MDA-MB-231 cells were washed twice with ice-cold PBS and lysed (buffer consisted of 40 mM HEPES pH 7.5, 120 mM NaCl, 1 mM EDTA, 1% Triton X-100, 0.1% SDS, 10% glycerol, 10 mM sodium pyrophosphate, 50 mM sodium fluoride, 1.5 mM sodium orthovanadate, and 1 mM PMSF, supplemented with a protease inhibitor cocktail) (Sigma-Aldrich, France). Cell lysates were then cleared by centrifugation (6,000 g, 10 min, 4°C) and stored at −80°C until analysis. Supernatants were collected and protein concentration was determined using the BCA assay (Sigma-Aldrich).

### Immunoprecipitation

Cell lysates were pre-cleared using matching antibody isotype and protein-G agarose beads (50% slurry in PBS, 1 h, 4°C). Cell lysates were then incubated with primary antibodies (2 h, 4°C) and complexes were precipitated with protein-G agarose beads (50% slurry in PBS, 2 h, 4°C) and eluted in Laemmli 2X buffer. Protein-G agarose beads were obtained from Millipore (France) and antibody isotypes from R&D Systems (France). The primary antibodies used in this study were: anti-HA (Roche, France) and anti-CD44 (Cell Signaling Technologies, Ozyme, France).

### Cell surface biotinylation assays

Cells were starved and chilled on ice, rinsed twice with ice-cold biotinylation buffer (PBS, 1 mM CaCl_2_, 0.5 mM MgCl_2_), and then incubated (30 min, 4°C) with a membrane-impermeable EZ-Link-Sulfo-NHS-LC-biotin (1 mg/mL; Thermo Scientific, Belgium) to label membrane proteins. Free biotin was quenched (0.1 M glycine, 30 min, 4°C) and washed twice with ice-cold biotinylation buffer. Cells were then incubated in pre-warmed media at 37°C with or without NGF. Cells were then incubated with 1 mM Dithiobis Succinimidyl Propionate (Thermo Scientific) dissolved in hybridization buffer (10 mM HEPES pH 7.5, 150 mM NaCl, 0.2 mM CaCl_2_ and 0.2 mM MgCl_2_) (30 min, 4°C) and then neutralized (20 mM Tris-HCl pH 7.5 and 150 mM NaCl) (30 min, 4°C). This incubation was followed by cell lysis and biotinylated proteins were then isolated by immobilization on streptavidin agarose resins (Thermo Scientific) (3 h, 4°C). Beads were washed three times (lysis buffer, 4°C) and eluted in Laemmli buffer (7 min, 95°C).

### Nano-LC-MS/MS Q-Star analysis

Peptidic digests were extracted from the 1-D gel band and nanoLC-nanoESI-MS/MS analyses were performed on an hybrid quadrupole time-of-flight mass spectrometer (Q-Star, Applied Biosystems, France) equipped with a nano-electrospray ion source coupled with a nano high pressure liquid chromatography system (LC Packings Dionex, France) as previously described [35].

### Western blot

Whole cell lysates or immunoprecipitated proteins were separated by SDS-PAGE electrophoresis and transferred to PVDF membranes (Westran® Clear Signal). The membranes were blocked with 5% milk or bovine serum albumin (BSA) in TBS-0.1% Tween20 (TBS-T) and subsequently immunoblotted overnight at 4°C. The membranes were then probed with horseradish peroxidase-conjugated secondary antibodies (Jackson Immunoresearch, Beckman Coulter, France) followed by SuperSignal West Pico Substrate (Thermo Scientific). Chemiluminescence was detected with a Fuji LAS-4000 luminescent image analyzer. The antibodies used in this study were as follows: anti-actin (Sigma-Aldrich), anti-HA (Covance, Eurogentec, France), anti-pan-CD44, anti-phosphoTrkA (Tyr-674/675), anti-p115-RhoGEF, anti-ROCK1, anti-RhoA, anti-RhoC, anti-phosphoAkt (Ser-473), and anti-pan-Akt (Cell Signaling Technologies).

### Flow cytometry analysis of plasma membrane level TrkA and CD44

Cells were washed twice with ice-cold PBS and then incubated with isotype antibody-FITC (BD Biosciences), anti-TrkA antibody (Abcam), or anti-CD44-FITC antibody (BD Biosciences), (1 μg in 1% SVF, 30 min, 4°C). Levels of TrkA and CD44 were analyzed using flow cytometry and Summit 4.5 software (Beckmann Coulter, France).

### *In situ* proximity ligation assay (PLA)

Cells (10^4^ cells per well) were grown on acid-washed eight-well glass slides (Thermo Scientific) in EMEM plus 10% FBS for 24 h. After treatment, paraformaldehyde-fixed cells were incubated with 4% BSA (1 h, 20°C) followed by overnight incubation with primary antibodies [rabbit anti-HA, 1:50, (Sigma); rabbit anti-TrkA, 1:50, (Alomone, Israel); mouse anti-CD44, 1:200, (Cell Signaling Technology)]. PLA was performed as recommended by manufacturer instructions.

### siRNA

The siRNA sequences used (100 pmol for each transfection) were against CD44 (GUAUGACACAUAUUGCUUC) and p115RhoGEF (GCAGCUCUGAGAACGGCAA) (Eurogentec, France). siCTRL (scramble siRNA) were from Life technologies. siRNA transfection was performed using INTERFERin^TM^ according to the manufacturer's instructions (Polyplus transfection, Ozyme, France).

### RhoGTPases activation assay

Cells were washed twice with ice-cold PBS, lysed in 50 mM Tris pH 7.4, 150 mM NaCl, 5 mM MgCl_2_, 0.1% Triton X-100, 1 mM DTT, supplemented with protease inhibitor cocktail (Roche). Cell lysates were prepared as described above. RhoA-GTP or RhoC-GTP levels were quantified as described previously [36].

### Cell invasion

Invasion assays were performed as previously described [8] in Boyden microchambers (BD Biosciences) with 8 μm pore size membranes.

### Clonogenic cell growth

Clonogenic assays were performed as previously described [37]. After siRNA transfection, 2,000 cells were seeded in 35 mm petri dishes. Colonies were stained with crystal violet [35] and colonies of at least 50 cells were counted after one week of culture.

### Tumor xenograft growth in immunodeficient mice

HA-TrkA MDA-MB-231 cells (3 × 10^6^) were subcutaneously injected into six-week old female SCID mice. Two weeks after cancer cells injection, mice were randomized into treatment groups (*n* = 7), and were treated a total of three times at 3 day intervals. Lestaurtinib (Calbiochem) was suspended in vehicle (40% polyethylene glycol 1000, 10% povidone C30 and 2% benzyl alcohol in distilled water) and injected intraperitoneally. CD44 siRNA (7.5 μg/mouse) or scramble siRNA (7.5 μg/mouse) were delivered using *in vivo* jetPEI^®^ according to the manufacturer's instructions (Polyplus transfection) and injected subcutaneously near the tumor mass. Tumor volume was determined throughout the experiment by measuring the length (l) and width (w) and calculated as π/6 × l × w × (l + w)/2.

### Statistical analysis

Statistics were performed with GraphPad Prism 5.01 software. Quantitative variables were analyzed by one-way ANOVA with Bonferroni's post-test (*p* < 0.001). Data from the *in vivo* experiment were analyzed using the Mann-Whitney test.

## SUPPLEMENTARY METHODS, FIGURES


