# Supplementary material for: NGF-induced TrkA/CD44 association is involved in tumor aggressiveness and resistance to lestaurtinib
Source: Oncotarget. 2015 Mar 25;6(12):9807–19. doi: 10.18632/oncotarget.3227 (PMC4496399; doi:10.18632/oncotarget.3227)
Supplement: Supplementary file 1 [file oncotarget-06-9807-s001.pdf]

SUPPLEMENTARY METHODS

Origin of other cancer cell lines

CAL 27, CAL 33, SQ20B and FaDu human head and neck cancer cells were obtained from Dr S Meignan (Centre Oscar Lambret, Lille). DU 145 and PC-3 prostate cancer cells, MCF-7 and SkBr3 breast cancer cells and HT-29 colon cancer cells were acquired from American Type Culture Collection (ATCC, Manassas, VA, USA) and were maintained in Eagle's Minimal Essential Medium (EMEM; MCF-7), Dulbecco's Modified Eagle's Medium (DMEM; CAL 27, CAL 33, SQ20B and HT-29 cells), RPMI 1640 medium (DU 145, PC-3 and FaDu cells) or Mc Coy's5A (SkBr3 cells) (Invitrogen Corporation, France) supplemented with 10% inactivated FBS (Fetal Bovine Serum) (Hyclone, France), 2 mM L-glutamine, 1% non-essential amino acids, 40 UI/ml penicillin, 40 µg/ml streptomycin, 50 µg/ml gentamycin and ZellShield™ (1X, Biovalley, France) at 37°C in 5% CO_2-humidified atmosphere. All experiments were carried out with cells of passage number less than 20.

Table identifiers and GO (Gene Ontology) annotation

The different categories of biological processes were manually associated to each protein. The terms "metabolism", "transport", "cell cycle", "apoptosis", "migration/invasion", "RNA processing", "protein processing" and "Others" were queried for the biological processes associated to each protein. Only proteins with Mascot score ≥ 40 and number of peptides > 1 were presented in the pie charts (Supplementary Figure S1). All commons proteins shared by both conditions (with or without NGF for a 30 min incubation period) in each purification technique were withdrawn of Table 1 and Supplementary Figure S1.

SUPPLEMENTARY FIGURES

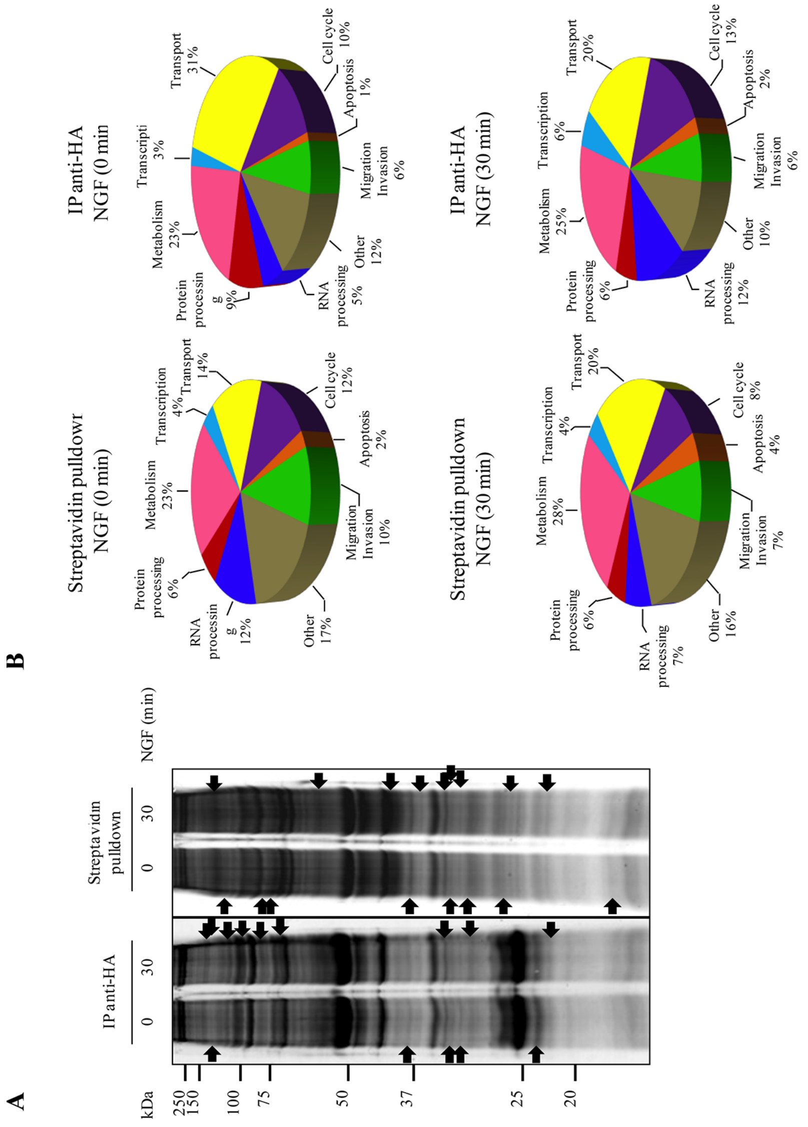

Supplementary Figure S1: Proteomic analysis of NGF/TrkA signaling. (A) Location of excised bands on SDS-PAGE gel (Arrows). HA-TrkA MDA-MB-231 cells were biotinylated and treated or not with NGF for 30 min. HA-TrkA immunoprecipitation or streptavidin pull-down was then performed on cell lysates (15 mg per condition) and the eluted proteins were separated by SDS-PAGE. Differential band cutting (indicated by arrows) was performed after Colloidal Coomassie Blue staining and the excised bands were trypsin-digested and analyzed by mass spectrometry as described in Materials and Methods. **(B)** Distributions of Gene Ontology annotations. Proteins identified by mass spectrometry were classified according to biological process wherein they are involved.

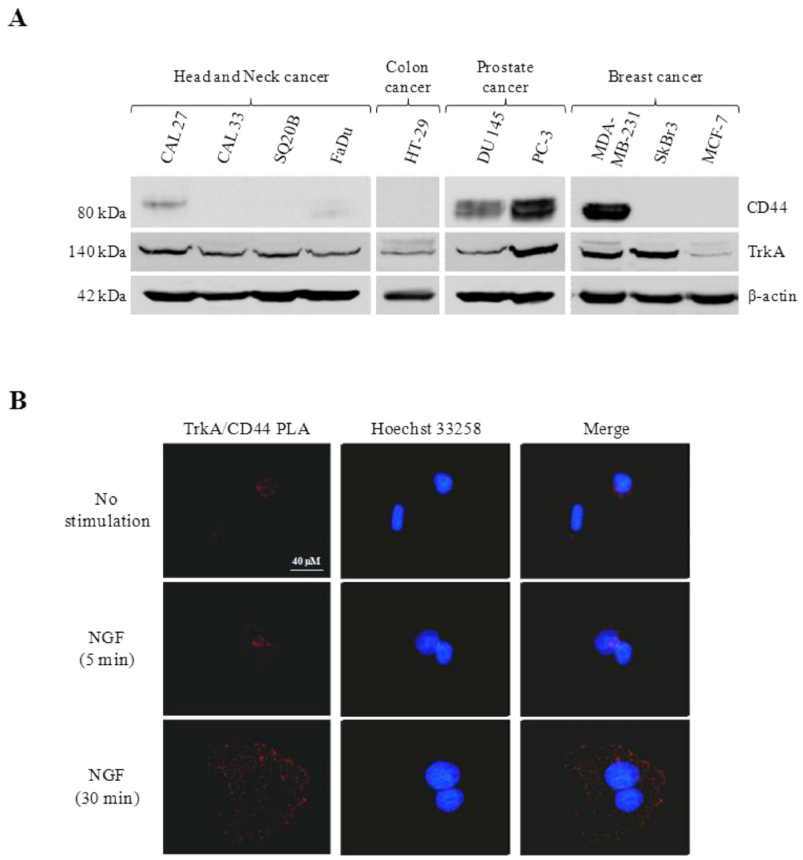

Supplementary Figure S2: Involvement of TrkA/CD44 association in other cancers. (A) Expression of TrkA and CD44 receptors in several cancer cell lines. Whole cell lysates from cancer cells were immunoblotted using CD44 and TrkA antibodies. Equal loading was checked with β-actin antibody. **(B)** NGF induced direct interaction between TrkA and CD44 in PC-3 prostate cancer cells. Cells were treated with NGF (5 and 30 min) and prepared for PLA experiment as described in Materials and Methods. Interactions between TrkA and CD44 were detected as red spots.

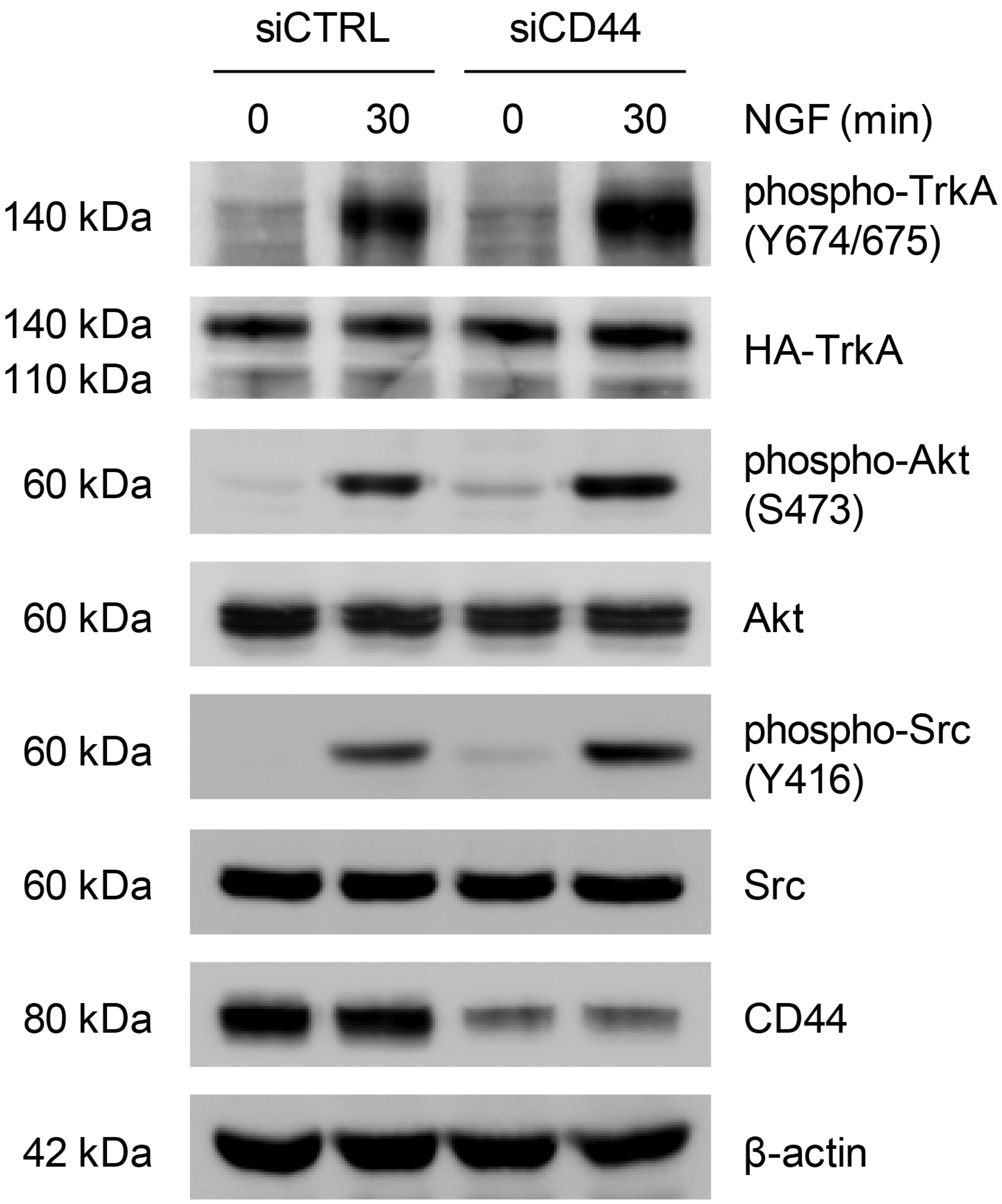

Supplementary Figure S3: Inhibition of CD44 expression does not affect NGF-induced TrkA activity and canonical TrkA pathways. MDA-MB-231 cells overexpressing HA-TrkA were transfected with siRNA directed against CD44 (siCD44), or control siRNA (siCTRL). Cells were then stimulated with NGF (5 or 30 min). Phosphorylations of TrkA, Akt and Src were assessed by using phospho-specific antibodies. Equal loading was verified with HA, Akt and Src antibodies.

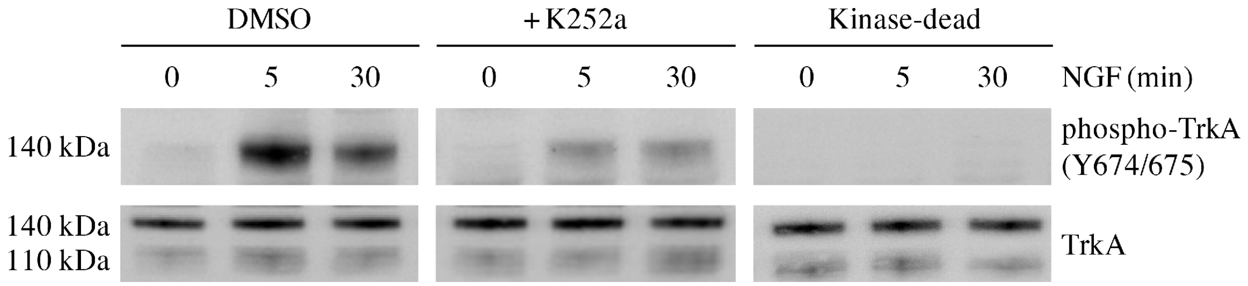

Supplementary Figure S4: Inhibition of TrkA phosphorylation. HA-TrkA MDA-MB-231 cells incubated with DMSO or with K252a, and HA-TrkA kinase-dead MDA-MB-231 cells were treated with NGF (5 or 30 min). TrkA phosphorylation was assessed by immunoblotting with phospho-specific antibody. Equal loading was verified with HA antibody.
